# How elephants beat cancer

**DOI:** 10.7554/eLife.21864

**Published:** 2016-10-31

**Authors:** Stephen J Gaughran, Evlyn Pless, Stephen C Stearns

**Affiliations:** Department of Ecology and Evolutionary Biology, Yale University, New Haven, United States; Department of Ecology and Evolutionary Biology, Yale University, New Haven, United States; Department of Ecology and Evolutionary Biology, Yale University, New Haven, United Statesstephen.stearns@yale.edu

**Keywords:** African elephant, hyrax, armadillo, aardvark, Asian elephant, Other

## Abstract

Elephants have significantly reduced their risk of cancer by duplicating an important gene called *TP53*.

**Related research article** Sulak M, Fong L, Mika K, Chigurupati S, Yon L, Mongan NP, Emes RD, Lynch VJ. 2016. *TP53* copy number expansion is associated with the evolution of increased body size and an enhanced DNA damage response in elephants. *eLife* **5**:e11994. doi: 10.7554/eLife.11994

Cancer is a genetic disease in which cells divide uncontrollably. Some of the mutations that cause cancer are inherited, but most are the results of mistakes made when DNA is copied during cell division. By the time a person reaches adulthood, their DNA will have been copied about 30 trillion times, and each of these events could result in a cancer-causing mutation (Frank, 2010). Human cells also continue to replicate their DNA and divide throughout adult life: this is particularly true in the gut, lungs, skin and bone marrow, and again each cell division comes with the risk of cancer.

Since large, long-lived organisms experience more cell divisions than small, short-lived ones, they have a greater chance of accumulating cancer-causing mutations. Indeed, models suggest that if elephants and whales had the same risk of cancer per cell division as humans they could not exist (Figure 1). Instead, they would all die of cancer at a young age (Caulin and Maley, 2011). Clearly elephants and whales do exist, and neither of them have unusually high rates of cancer. This puzzle is referred to as Peto’s Paradox (Peto et al., 1975), and it hints that large-bodied animals must have mechanisms to compensate for experiencing so many cell divisions. Recently, two groups of researchers set out to discover how elephants evolved to prevent or suppress cancer, and both arrived at a single gene – *TP53*.

**Figure 1.**
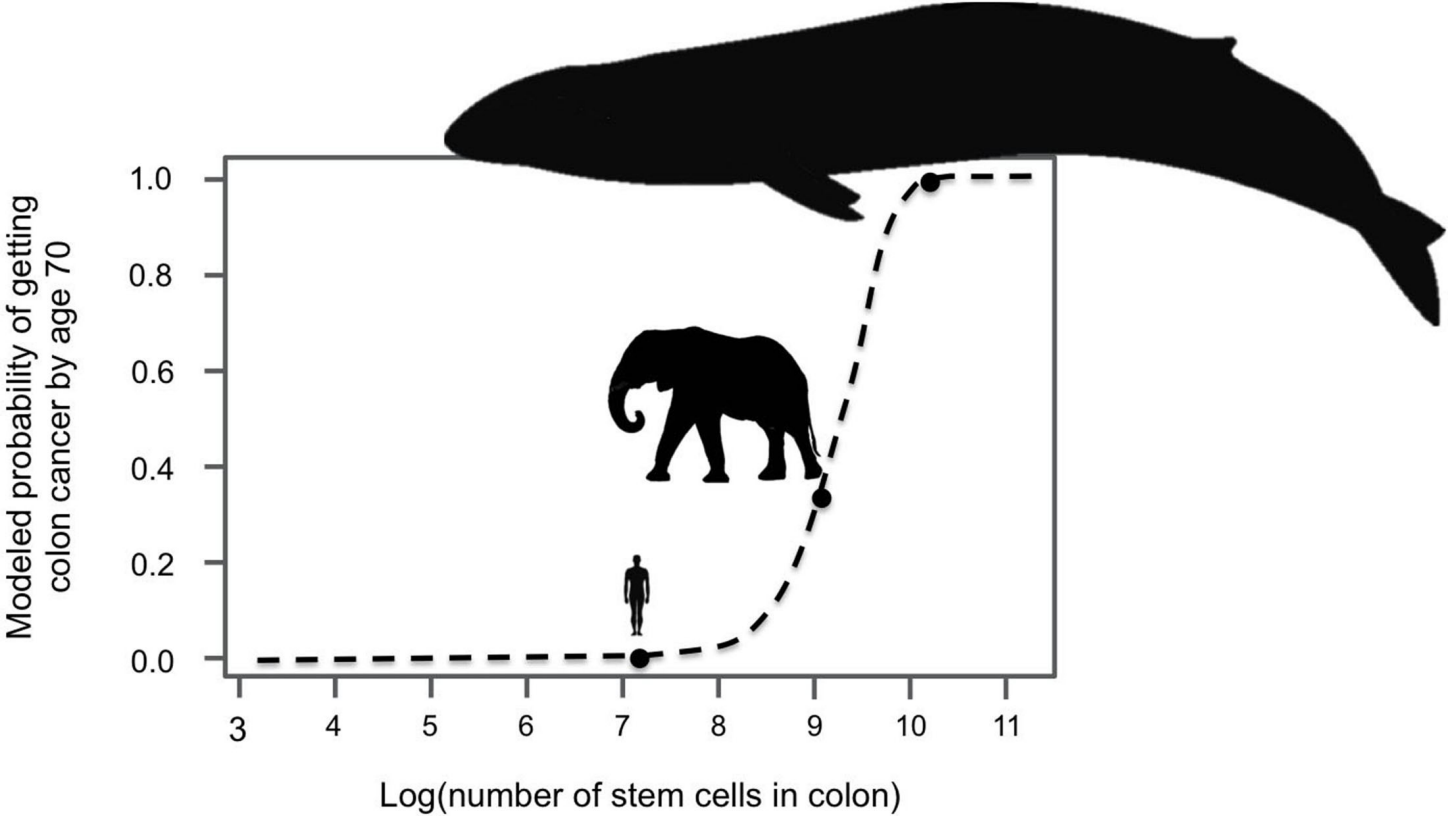
Large-bodied animals have much lower rates of cancer than models predict. Based on data on number of cell divisions and mutation rate, a model estimates that larger animals with larger colons should have a much higher risk developing colon cancer by age 70 (dashed line). This predicts a probability of less than 1% for humans, which matches reported incidence statistics in the UK (Cancer Research UK). However, although the model estimates much higher probabilities for large-bodied animals such African elephants and blue whales, cancer risk is actually much lower in elephants than in humans. Sulak et al. suggest that elephants have evolved to have this significantly reduced risk of cancer by replicating the tumor suppressor gene *TP53*. Whales appear to have evolved other solutions, which remain unknown. This model and figure are adapted from Caulin and Maley (2011).

In humans, the *TP53* gene protects against cancer, and mutations that prevent the gene from working are behind many cancers in adults (Hollstein et al., 1991). Moreover, children who inherit a mutated copy of *TP53* develop a variety of childhood cancers and have a lifetime risk of cancer that is 73% in men and nearly 100% in women (McBride et al., 2014).

Last year, Joshua Schiffman, Lisa Abegglen (both from the University of Utah School of Medicine) and colleagues reported a number of interesting results on *TP53* genes in elephants (Abegglen et al., 2015). First they confirmed that an elephant’s cancer risk is about 2–5 times lower than a human’s; they then went on to show that elephants actually have 20 copies of *TP53*. Abegglen et al. also noted that while one of the elephant’s *TP53* genes was comparable to those in other mammals, the other 19 were slightly different. Most genes contain a mix of protein coding sections (which are called exons) and non-coding sections (called introns). Typically, introns are removed after a gene has been transcribed into messenger RNA but before it is translated into a protein. However, all but one of the *TP53* genes in elephants lacked true introns. This indicates that the 19 extra *TP53* genes likely originated when an edited RNA molecule, which had had its introns removed, was converted back to DNA. Genes with this kind of history are known as “retrogenes”. Still, Abegglen et al. did not establish when these extra copies of *TP53* first evolved.

One way that the *TP53* gene protects against cancer is by causing cells with damaged DNA (which is likely to contain cancer-causing mutations) to commit suicide, via a process known as apoptosis. Abegglen et al. exposed elephant cells to ionizing radiation (which causes DNA damage) and found that they were twice as likely to undergo apoptosis as cells from healthy humans. However, based on this pair-wise comparison, it was not clear whether the elephant cells are more prone to apoptosis, or if human cells are relatively insensitive to DNA damage. Abegglen et al. also did not establish how and to what extent the *TP53* retrogenes were transcribed in elephant cells.

Now, in eLife, Vincent Lynch and colleagues – including Michael Sulak as first author – report answers to many of the remaining open questions about *TP53* in elephants (Sulak et al., 2016). First, Sulak et al. searched 61 genomes of animals ranging from aardvarks to whales for *TP53* genes and retrogenes. Some of these animals – such as manatees and the rock hyrax – had only a few *TP53* retrogenes, whereas others had multiple copies of *TP53* retrogenes. By mapping the data onto a phylogenetic tree, Sulak et al. showed that the number of *TP53* genes had increased as body size increased in the lineage that led to elephants.

Sulak et al. – who are based at the University of Chicago, the University of Nottingham and Weill Cornell Medical College – then experimentally established several key points. They confirmed that some of the *TP53* retrogenes are transcribed and translated in elephant tissue, and that these transcripts give rise to multiple forms of the proteins. Also, elephant cells up-regulated *TP53* signaling and induced apoptosis in response to lower levels of DNA damage (from drugs and radiation) than cells from other mammals. This indicates that elephant cells are especially sensitive to DNA damage and more prone to apoptosis. Next, Sulak et al. showed that elephant cells need the retrogenes for their enhanced apoptosis response. Finally, adding the same retrogenes to mouse cells made these cells more sensitive to DNA damage too. Cell division despite DNA damage is a hallmark of cancer, and so Sulak et al. concluded that elephants had likely solved Peto’s Paradox (at least in part) by enhancing *TP53* signaling, a feat that they achieved by duplicating the *TP53* gene.

The findings of Sulak et al. – which were first reported in a preprint on bioRxiv – answer many of the questions posed following the work of Abegglen et al. However, they also leave several issues unresolved. First, it is not clear if the retrogenes themselves lead to the increased apoptosis response, or whether other actors are in play. Second, does having multiple copies of *TP53* impose costs and, if so, to what extent can elephants avoid them? Moreover, Sulak et al. did not find multiple copies of *TP53* in whales; what other mechanisms suppress or prevent cancer in these creatures? Finally, can such insights be translated into cancer treatments in the clinic?

Results like these showcase how evolutionary thinking can illuminate medical problems, especially when combined with experiments that check assumptions and establish causation. Comparative evolutionary biology suggested the paradox; phylogenetic methods revealed that gene copy number and body size increased together; and genetic experiments confirmed that the key causal links exist. Such combinations of evolutionary questions and molecular methods should rapidly advance cancer research, and may one day tell us how to prevent cancers if possible and treat them if necessary.
